# Estimation of adequate setup margins and threshold for position errors requiring immediate attention in head and neck cancer radiotherapy based on 2D image guidance

**DOI:** 10.1186/1748-717X-8-212

**Published:** 2013-09-10

**Authors:** Mika Kapanen, Marko Laaksomaa, Tapio Tulijoki, Seppo Peltola, Tuija Wigren, Simo Hyödynmaa, Pirkko-Liisa Kellokumpu-Lehtinen

**Affiliations:** 1 Department of Oncology, Tampere University Hospital (TAUH), Teiskontie 35, PO BOX 2000, FI-33521 Tampere, Finland

**Keywords:** Radiotherapy, Head and neck cancer, Setup margins, Adaptive replanning, Image guidance

## Abstract

**Background:**

We estimated sufficient setup margins for head-and-neck cancer (HNC) radiotherapy (RT) when 2D kV images are utilized for routine patient setup verification. As another goal we estimated a threshold for the displacements of the most important bony landmarks related to the target volumes requiring immediate attention.

**Methods:**

We analyzed 1491 orthogonal x-ray images utilized in RT treatment guidance for 80 HNC patients. We estimated overall setup errors and errors for four subregions to account for patient rotation and deformation: the vertebrae C1-2, C5-7, the occiput bone and the mandible. Setup margins were estimated for two 2D image guidance protocols: i) imaging at first three fractions and weekly thereafter and ii) daily imaging. Two 2D image matching principles were investigated: i) to the vertebrae in the middle of planning target volume (PTV) (MID_PTV) and ii) minimizing maximal position error for the four subregions (MIN_MAX). The threshold for the position errors was calculated with two previously unpublished methods based on the van Herk’s formula and clinical data by retaining a margin of 5 mm sufficient for each subregion.

**Results:**

Sufficient setup margins to compensate the displacements of the subregions were approximately two times larger than were needed to compensate setup errors for rigid target. Adequate margins varied from 2.7 mm to 9.6 mm depending on the subregions related to the target, applied image guidance protocol and early correction of clinically important systematic 3D displacements of the subregions exceeding 4 mm. The MIN_MAX match resulted in smaller margins but caused an overall shift of 2.5 mm for the target center. Margins ≤ 5mm were sufficient with the MID_PTV match only through application of daily 2D imaging and the threshold of 4 mm to correct systematic displacement of a subregion.

**Conclusions:**

Adequate setup margins depend remarkably on the subregions related to the target volume. When the systematic 3D displacement of a subregion exceeds 4 mm, it is optimal to correct patient immobilization first. If this is not successful, adaptive replanning should be considered to retain sufficiently small margins.

## Background

Radiotherapy (RT) of head and neck cancers (HNC) is one of the most challenging RT procedures [1]. This is because the target and risk tissues have very complex shape and are very close to each other. In addition, the treatment area is subject to anatomical deformations mainly caused by tumour regression/progression and patient weight loss. Detection of soft tissue deformations requires 3D imaging such as kV or MV based computed tomography (CT) or magnetic resonance imaging (MRI) [2]. Fortunately, current onboard imaging technology has enabled acquisition of kV or MV tomography images in treatment situation for on-line verification of target and risk organ localization [3] instead of indirect information based on bony landmarks visible in planar 2D images. Unfortunately, the tomographic 3D imaging requires much more resources than the 2D imaging and causes extra radiation exposure for the patients setting potential limits for its frequent use. As a consequence, routine patient setup verification is mostly based on 2D imaging and 3D imaging is applied less frequently to control the relevant soft tissues and their relation to the bony landmarks [2]. The frequency of the 3D imaging, however, is usually optimized for changes in soft tissues and may vary remarkably between the RT centres. Criteria to screen patients needing immediate attention due to changes in patient setup and posture should be established but the topic has not been addressed properly in the literature.

When the 2D image guidance is applied more frequently than the 3D imaging, residual setup errors are predominantly determined by the 2D position verification and the setup margins should be confirmed sufficient for such procedure. Small isotropic margins from 3 to 5 mm have been considered sufficient by assuming a rigid target [4,5]. Tissue deformations, however, may require different margins for the subregions within the target volume. Such data have been published for the bony landmarks based on onboard cone beam CT (CBCT) imaging [6-8] but to the best of our knowledge not for the 2D kV imaging. 2D kV and CBCT based alignments have been found to correlate highly but different margins may be required [9]. Sufficient setup margins depend always on the image guidance protocol used [10,11] and the quality of patient immobilization. Therefore, several studies applying different protocols and immobilization techniques would be beneficial.

The purpose of this study was to estimate how large margins are needed in the HNC RT when the 2D kV imaging is used for frequent setup verification. The target volume was described by the most relevant bony landmarks visible in those images such as the vertebrae C1-2, C5-7, the occiput bone and the mandible. We estimated a threshold for position errors of the subregions requiring immediate attention based on two new principles using clinical data of position errors. Previously, patient replanning rate has been applied [6,12]. We suggested actions when the threshold obtained was exceeded based on our clinical experience. The goal was to retain the commonly used isotropic margin of 5 mm sufficient for all the subregions. Moreover, we determined the contribution of observer variation in 2D image online matching which has not been comprehensively investigated in the literature. We determined the displacements of the subregions in actual treatment situation. Setup margins were calculated for daily and weekly image guided RT (IGRT) protocols combined with two image matching principles. The investigation was limited to errors related to image matching.

## Methods and material

### Patient group and clinical IGRT protocol

We analyzed retrospectively 80 consecutive head-and-neck cancer patients treated with external radiotherapy at prescribed doses of 60–70 Gy, irrespectively of tumour stage and location. The average age was 63 years (range from 39 to 89 years). Candor head and neck plate with 5-point C-frame (Candor, Gislev, Denmark) was used for patient fixation. The device included head cushion, 5-point thermoplastic mask but no mouth block. CT imaging for treatment planning was performed with either Philips Brilliance Big Bore (Philips Medical Systems, Eindhover, The Netherlands) or Toshiba Aquilion LB (Toshiba Medical System, Tokyo, Japan) at 120 kV using a slice thickness of 3 mm. The patients were treated with 7-field IMRT technique using 6 MV photon beams of Clinac 2300 iX (Varian Medical Systems, Palo Alto, CA). Image guidance was carried out using orthogonal kV-images acquired with an onboard imaging system (OBI) at 100 and 70 kV for anterior-posterior and lateral images, respectively. For 50 patients, the image guidance was performed online at 3 first treatment fractions and weekly thereafter (the weekly IGRT protocol) and 30 patients were imaged daily (the daily IGRT protocol). Patient setup was corrected for translational shifts without tolerance. If the setup error was ≥ 3 mm in any direction, image guidance was repeated in the subsequent treatment fraction. If systematic (average) patient setup error of ≥ 3 mm was detected in the first three fractions or thereafter at least in two successive fractions, patient setup marks were corrected for this error. The setup marks were corrected also for the average vertical reading of the first three fractions without tolerance. The corrections of patient setup were taken into account in the calculation of the setup errors.

### Investigated matching principles

**Figure 1:**
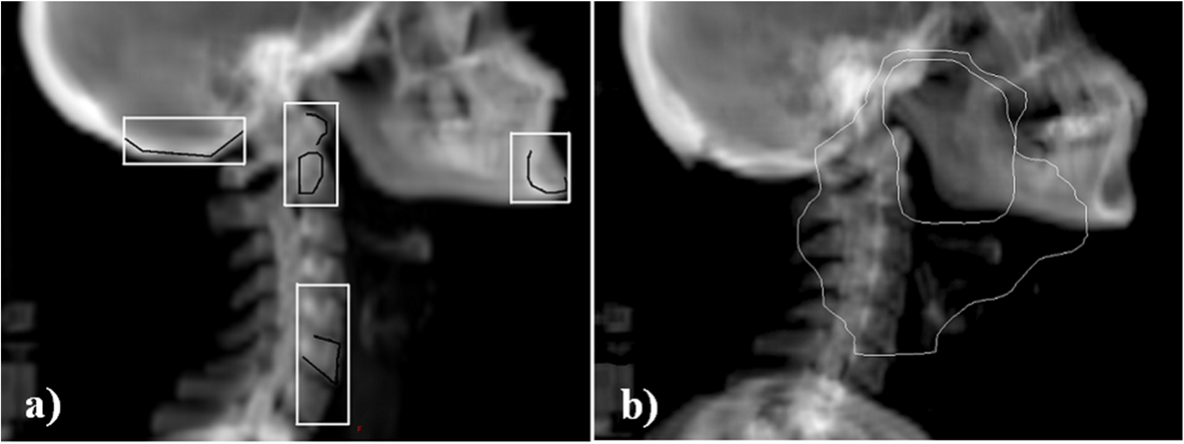
**Selection of bony landmarks related to the target volumes in HNC RT. a)** Selection of four subregions (white boxes) to measure the combined effect of rotation, mutual movement and shape changes of the bony landmarks. Image matching for these areas was based on well defined structures (indicated in black) in the vertebrae C1-2 and C5-7, the mandible and the occiput bone. **b)** Example of large and small CTV volumes in tonsilla carcinoma (outer and inner lines) with prescribed doses of 54 and 60 Gy, respectively. The lower dose volumes may extend to all four subregions while the higher dose volumes (receiving 60 Gy or more) typically covers only one or two of the subregions.

The most important bony landmarks related to the clinical target volumes (CTVs) were divided into four subgroups to account for the combined effect of rotation, mutual position changes and shape changes of these structures. The chosen subregions were the vertebrae C1-2, C5-7, the mandible and the occiput bone illustrated in Figure 1. For 90% of the patients CTV extended to all the four subregions. Two different image matching principles were investigated retrospectively. The first match was done to the vertebrae in the middle of PTV (MID_PTV) (the combination of multiple PTVs with SIB technique). The other was done by minimizing maximal position error for the bony landmarks in the four subregions (MIN_MAX) [12]. These manual reference matches were performed by two experienced observers to minimize contribution of subjective variation.

### Residual position errors of the reference location

The reference location for the image matching was the vertebrae in the middle of PTV maximizing the coverage of PTV in the treatment fields. In 98% of the cases the reference site was the vertebrae C3-4. We determined residual position errors of this location when the setup corrections were done according to the investigated image matching principles combined with the above setup correction protocol. This was done for 908 images acquired from the 30 patients imaged daily. The results for daily IGRT were derived directly while the presented weekly IGRT protocol was simulated by omitting couch corrections on the days when image guidance was not performed according to the protocol.

### Estimation of observer related variation

We determined the difference of the reference location between the reference MID_PTV match and an on-line MID_PTV match performed by 25 experienced radiation technologists in actual treatment situation. The results of 583 images from 50 patients were analyzed. Both the systematic and random components were determined. Observer related variation in the MIN_MAX match was determined offline for the same data and group of technologists. Since the position error of the reference location reflects changes merely in patient setup, error for that location was estimated also including the observer variation in the image matching. This was done by adding the observer errors obtained in squares assuming independent gaussian type errors (separately for the systematic and random components).

### Residual position errors of the subregions

Differences between the positions of the subregions in the reference images and in the treatment verification images were determined for the two matching principles. The results of the MID_PTV online matches and the MIN_MAX offline matches described above were analyzed. The obtained shifts include translations of the bony landmarks due to their rotation, mutual movement and shape changes, and observer related variation in choosing an optimal image match site. The results provide directly local residual errors for daily imaging because the variation in patient posture exceeding the tolerance is corrected by the observer. The results for the weekly IGRT protocol were obtained by adding the position error of the reference location in squares. This was done separately for the systematic and random components. Rotations were not explicitly determined since they cannot be automatically corrected.

Correlation coefficients (ρ) were calculated for the displacements of the subregions with respect to their planned locations in the reference images for the both matching principles. This was chosen because correlation between the subregion movements with respect to a fixed reference structure (such as the vertebrae C1-3) have been published earlier [6].

### Determination of adequate margins

Setup margins were estimated for the whole target volume and for the individual subregions assuming that these structures act as rigid objects. We used van Herk’s formula [13]:

(1) m=2.5Σ+0.7σ

where Σ is systematic setup error and σ is random setup error both given as one standard deviation. In this study, Σ is defined conventionally as a standard deviation of average errors calculated for individual patients weighted by the number of acquired images. σ is calculated as root-mean-square value over all displacements around the systematic setup errors as presented in [14]. We calculated the setup margins for the reference location with and without observer related errors, and for daily and weekly IGRT protocols. The margins for the subregions include always observer errors as described above. We estimated also empirical 3D margins by calculating 90 and 95% upper limits for pooled 3D geometric errors.

### Estimation of threshold for position errors

We determined upper limit of systematic (average) 1D and 3D displacements for each subregion to screen potentially clinically meaningful treatment localization errors. The limit was determined with two previously unpublished methods by retaining an isotropic margin of 5 mm sufficient for each of the investigated subregions: i) the systematic errors were corrected for the patients starting from the patient with the largest systematic 3D error and by recalculating the margins for that subregion, ii) the threshold was determined more theoretically by adjusting *m* = 5 mm in equation (1) and by calculating maximal allowed systematic error (term 2.5Σ) using the empirical σ values obtained.

## Results

### Summary of residual setup errors

**Figure 2:**
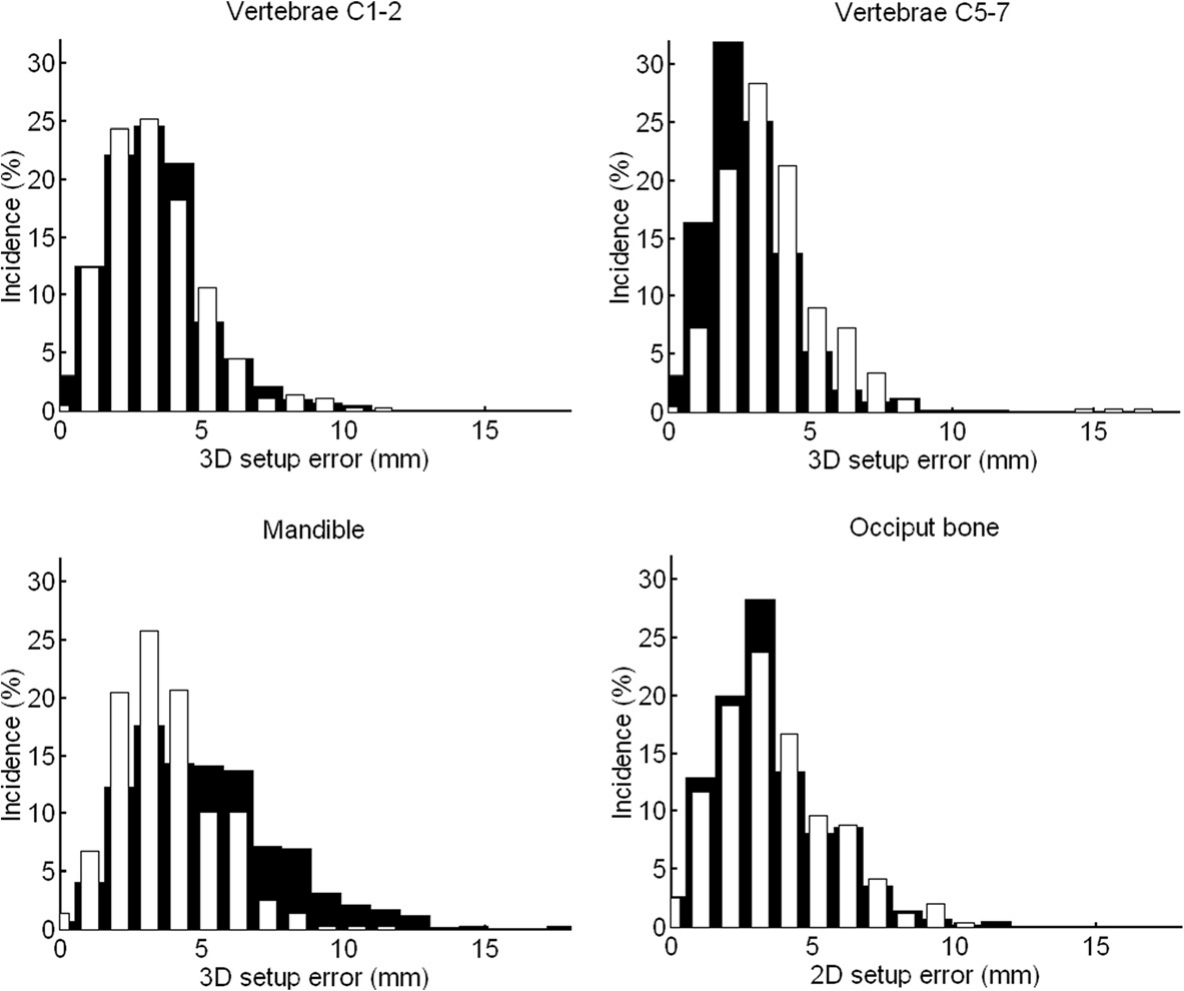
**3D setup errors for the four subregions.** The black bars present the results of the MID_PTV match, while the white bars demonstrate the results of the MIN_MAX match. The errors present daily imaging and include observer variation. Note that 2D errors are given for the occiput bone.

Position errors of different sources and regions are summarized in Table 1. The errors of the subregions present the situation with daily imaging. For the presented weekly IGRT protocol, position error of the reference location should be added in squares to these errors. 3D setup errors are shown in Figure 2. The weekly IGRT protocol required that approximately 1/3 of the treatment fractions were imaged in practise. In each category the average errors were very small (< 1 mm) except 1.7 mm obtained for the mandible in vertical direction (anteriorly) with the MID_PTV match. Incidence of large observer related deviations ≥ 5 mm was 2.0/0.8/1.0% in vertical/longitudinal/lateral direction, respectively.

**Table 1:** Error components for the presented setup correction protocol in different directions (mm)

**Structure**	**Systematic error Σ (1 SD)**			**Random error σ (1 SD)**		
	**AP/ vertical**	**CC/ longitudinal**	**LAT/ lateral**	**AP/ vertical**	**CC/ longitudinal**	**LAT/ lateral**
Rigid target^1^	0.8 (1.1)	0.9 (1.3)	0.7 (0.7)	1.7 (1.3)	1.6 (1.6)	1.6 (1.2)
Observer variation	0.9 (1.0)	0.8 (1.0)	0.9 (0.9)	1.4 (1.4)	1.5 (1.6)	1.6 (1.6)
C1-2^2^	2.0 (1.6)	1.0 (1.4)	1.6 (1.3)	1.8 (1.4)	1.3 (1.6)	1.6 (1.5)
C5-7^2^	1.5 (1.8)	1.0 (1.5)	1.2 (1.2)	1.4 (1.6)	1.8 (1.9)	1.7 (1.6)
Mandible^2^	2.9 (1.5)	2.7 (1.5)	1.6 (1.3)	2.3 (1.7)	2.4 (1.9)	1.9 (1.6)
Occiput bone^2^	2.4 (2.2)	1.5 (2.0)	-	2.3 (1.9)	1.9 (1.7)	-

^1^Errors of the reference location for weekly IGRT without observer related variation.

^2^Errors with daily imaging including observer errors.

The results are given for the two matching principles as MID_PTV (MIN_MAX).

### Residual errors of the subregions

With the MID_PTV match, the mean ± SD (maximum) of systematic 3D errors (with respect to the planned location) for the vertebrae C1-2, C5-7 and the mandible were 2.5 ± 1.2 mm (5.7 mm), 2.0 ± 1.0 mm (5.2 mm) and 4.0 ± 1.9 mm (8.8 mm), respectively. With the MIN_MAX match, the corresponding 3D errors were 2.4 ± 1.2 mm (6.0 mm), 2.7 ± 1.3 mm (6.4 mm) and 2.7 ± 1.1 mm (5.3 mm), respectively. The percentages of cases exceeding the obtained threshold of 4 mm were 10, 2 and 38%, respectively. With the MIN_MAX match, the corresponding percentages were 6, 9 and 9%, respectively. For the occiput bone the percentages were 18 and 24% with the MID_PTV and MIN_MAX matches, respectively. Systematic position errors between the four subregions and their mutual correlations are given in Table 2. The main weaknesses of the used patient immobilization were patient rotation about lateral axis and large mandible movement.

**Table 2:** Systematic change in distance between the subregions due to rotation and shape changes of the bony landmarks

**Subregions**	**Practical importance**	**AP/ vertical**	**CC/ longitudinal**	**LAT/ lateral**
C1-2 and C5-7	Rotation of the vertebrae	3.1 mm	1.2 mm	2.3 mm
		ρ = −0.34 (−0.21)	ρ = 0.55 (0.82)	ρ = −0.14 (−0.06)
C5-7 and occiput bone	Rotation of patient	3.4 mm	1.5 mm	-
		ρ = −0.34 (−0.26)	ρ = 0.34 (0.63)	
C1-2 and occiput bone	Orientation of vertebrae and skull base	1.9 mm	1.3 mm	-
		ρ = 0.69 (0.60)	ρ = 0.55 (0.66)	
Mandible and occiput bone	Nod movement of the head	2.6 mm	4.0 mm	-
		ρ = 0.42 (0.37)	ρ = −0.27 (−0.27)	
Mandible and C1-2	Independent movement of mandible	2.2 mm	2.9 mm	1.5 mm
		ρ = 0.61 (0.30)	ρ = 0.12 (−0.22)	ρ = 0.62 (0.57)

Also correlation coefficients (ρ) for the position errors are given for MID_PTV and (MIN_MAX) image matches.

With 8 patients having the largest systematic setup errors (> 5 mm) for the subregions, visually clear time trend (and strong correlation between the position error and time) was found only for 2 patients (correlation of error with time ρ ≈ 0.8). For 6 out of these 8 patients, the large systematic position error was detected already during the first treatment week.

### Threshold of position errors

Empirically determined threshold for 3D systematic error was 4.3 mm (range from 4.1 to 4.5 mm) and 4.0 mm (range from 3.9 to 4.2 mm) for the MID_PTV and MIN_MAX match, respectively. Theoretically estimated threshold was 3.7 mm (range from 3.3 to 4.1 mm) and 3.8 mm (range from 3.6 to 4.0 mm), respectively.

### Adequate anisotropic margins

The margins required for different areas are presented in Table 3. The MIN_MAX match caused an overall shift of the target center since it corrects mainly the displacement of the mandible. Empirically determined 3D margins based on 90% confidence for the vertebrae C1-2, C5-7 and the mandible were 5.5, 4.6 and 8.7 mm, respectively, with the MID_PTV match and daily imaging, while with the MIN_MAX match, the corresponding margins were 5.4, 6.0 and 6.2 mm, respectively. The corresponding empirical 3D margins based on 95% confidence were 6.6, 5.5 and 10.3 mm, respectively, with the MID_PTV match, while with the MIN_MAX match, the margins were 6.2, 7.1 and 6.8 mm, respectively.

**Table 3:** Required setup margins (mm) for the presented weekly IGRT protocol in different directions without an action level for position errors of the subregions

**Structure**	**MID_PTV**				**MIN_MAX**			
	**AP/ vertical**	**CC/ longitudinal**	**LAT/ lateral**	**Mean 3D**	**AP/ vertical**	**CC/ longitudinal**	**LAT/ lateral**	**Mean 3D**
Rigid target^1^	3.1 (0)	3.3 (0)	2.7 (0)	3.1 (0)	3.8^4^ (0^4^)	4.4^4^ (0^4^)	2.7^4^ (0^4^)	3.6^4^ (0^4^)
Rigid target^2^	4.5 (3.2)	4.5 (3.1)	4.4 (3.4)	4.5 (3.2)	5.0^4^ (3.3^4^)	5.4^4^ (3.5^4^)	4.3^4^ (3.4^4^)	4.9^4^ (3.4^4^)
C1-2^2^	7.0 (6.2)	4.8 (3.4)	5.8 (5.1)	5.9 (4.9)	6.4 (5.1)	6.4 (4.6)	5.1 (4.3)	6.0 (4.7)
C5-7^2^	5.7 (4.7)	5.0 (3.8)	4.9 (4.1)	5.2 (4.2)	6.8 (5.6)	6.8 (5.1)	4.9 (4.0)	6.1 (4.9)
Mandible^2^	9.6 (8.9)	9.2 (8.5)	6.1 (5.3)	8.3 (7.6)	6.3 (5.0)	6.9 (5.2)	5.1 (4.3)	6.1 (4.8)
Occiput bone^2^	8.3 (7.6)	6.1 (5.1)	-	7.2^3^ (6.3^3^)	7.8 (7.3)	7.6 (6.1)	-	7.7^3^ (6.7^3^)

^1^Without observer errors, ^2^with observer errors, ^3^2D margins for the occiput bone, ^4^the MIN_MAX match shifted the reference location of the target by 2.5 ± 1.2 mm (3D mean ± SD) not included in the margins.

The results for daily IGRT are given in parenthesis.

Based on the results obtained for the action levels, a compromise threshold value of 4 mm was used to recalculate the margins. When large systematic 1D setup errors of ≥ 4 mm were corrected, the calculated margins were not remarkably reduced from those given in Table 3. The margins calculated by correcting systematic 3D errors of ≥ 4 mm are presented in Table 4. It can be seen that 5 mm isotropic margins are sufficient only by using the MIN_MAX image matching principle and by using daily imaging.

**Table 4:** Required setup margins (mm) for the presented weekly IGRT protocol in different directions with the action level of 4 mm for position errors of the subregions

**Structure**	**MID_PTV**				**MIN_MAX**			
	**AP/ vertical**	**CC/ longitudinal**	**LAT/ lateral**	**Mean 3D**	**AP/ vertical**	**CC/ longitudinal**	**LAT/ lateral**	**Mean 3D**
C1-2^1^	6.0 (5.1)	4.6 (3.1)	5.5 (4.7)	5.4 (4.3)	6.0 (4.7)	5.7 (3.6)	5.0 (4.2)	5.6 (4.2)
C5-7^1^	5.4 (4.3)	5.0 (3.7)	4.9 (4.1)	5.1 (4.0)	6.5 (5.3)	5.9 (3.8)	4.7 (3.8)	5.7 (4.3)
Mandible^1^	6.1 (5.2)	5.8 (4.7)	5.2 (4.4)	5.7 (4.8)	5.9 (4.5)	6.3 (4.5)	5.1 (4.3)	5.7 (4.4)
Occiput bone^1^	7.0 (6.2)	5.3 (4.1)	-	6.1^2^ (5.2^2^)	6.6 (5.5)	6.1 (4.2)	-	6.4^2^ (4.8^2^)

^1^includes observer errors, ^2^2D margins for the occiput bone.

The results for daily IGRT are given in parenthesis.

## Discussion

We determined adequate setup margins for routine setup verification in HNC RT based on 2D kV images and bony landmarks. The investigation was based on the most important landmarks (the vertebrae C1-2 and C5-7, the mandible and the occiput bone) clearly identified in orthogonal x-ray images. If position and deformation errors of the soft target and risk tissues with respect to the bones are already included in the CTV [15], the obtained margins around the CTV ensure that it receives 95% of treatment dose for 90% of the patients according to van Herk’s formula [13]. Our results suggest different considerations for adequate margins, image guidance and adaptive replanning assuming a rigid or a non-rigid target. The results were based on the displacements between the reference and treatment verification images. We excluded technical issues related to image voxel size, limited accuracy of couch shifts provided by the software and uncertainty of the couch movement. Also the contribution of intra fractional tissue movement was excluded. Two patient groups (daily IGRT *n* = 30 and weekly IGRT *n* = 50) were analyzed. The daily imaging was considered essential in the determination of position errors for the reference location. Larger patient group was chosen for the determination of these errors for the subregions.

### Rigid target

Our overall setup errors were within 0.5 mm with those reported for offline shrinking action level protocol [6] and daily portal imaging protocol [5]. Our results indicate that 5 mm isotropic setup margins are sufficient if the target can be assumed rigid as has most often been done in the literature [4,5]. We demonstrated that small 3 mm margins can be applied by adopting daily imaging [16] or if observer variation can be reduced. Small average errors (< 1 mm) between the reference match and observer MID_PTV matches suggest that the images have been matched usually quite close to the reference site in the vertebrae. The results implicate that it might be more optimal to reduce observer variation by harmonizing the choice of treatment localization instead of spending resources for daily imaging with rigid CTVs. This is consistent with a finding that imaging on every two or three day basis is sufficient [5]. We discovered that approximately half of the fractions were imaged in practise. This was needed to confirm the systematic nature of the setup errors.

Moderate correlations between the position errors of the subregions in vertebrae and the occiput bone suggest that these structures act only partly as one rigid area and mostly in cranio-caudal direction. Rotation, mutual movements and shape changes of the landmarks require larger margins if their contribution cannot be reduced. First attempt should be quality control and potential improvement of patient immobilization system and practise. We considered that poor quality of an individual case can be judged based on the results given in Table 1. Random setup error for a patient should not be much greater than the average random error obtained for the whole group. Otherwise corrective actions are needed for the fixation of that patient.

### Non-rigid and rotated target

When rotation, mutual movement and shape changes of the landmarks are taken into account, adequate margins can be chosen using the data given in Tables 3 and 4 depending on which subregions are relevant for the patient PTV. Generally it is not acceptable to use margins larger than 5 mm. Therefore, daily imaging and application of the action level of 4 mm for 3D systematic errors are needed for targets extending to all the four subregions. When this threshold is exceeded, immediate action is required to minimize the effect of systematic errors. Based on the results of this study, we introduced the action level obtained in our clinical practise. When this threshold was exceeded, we corrected the patient immobilization first (e.g. correction of rotation using thin plastic bars between the fixation baseplate and treatment couch), which was successful in most of the cases. If there was no improvement, we found optimal to perform an immediate onboard CBCT scan (or scans) to confirm the need of adaptive replanning. With the 3D IGRT acceptable compromise may be found for the soft tissues and some unnecessary replanning CT scans may be avoided. Furthermore, clear benefit of the 3D images is that they allow projection of planned dose distribution on the soft tissues facilitating the evaluation of the clinical importance of the position errors and the relevance of the bony landmarks. Different position errors, however, can not be expected for the bony landmarks with the 3D imaging. Future development of performing fast online dose calculation to calibrated CBCT images might be of a great benefit [3,17].

The MIN_MAX match seemed efficient only when the PTV includes the mandible and the obtained threshold was not used. The MIN_MAX match, however, tended to increase overall setup error by shifting the target center and required larger margins for the vertebrae than the MID_PTV match. Therefore, we prefer the MID_PTV match as also proposed in the literature [12].

Bony landmarks are clearly visible in all clinical image guidance systems (portal MV, kV, CBCT) and provide information of patient posture but not directly of the soft tissue deformations and tumour regression/progression. Therefore, regular 3D verification should be performed [2,3]. Especially the parotid glands tend to shrink and move toward patient midline during the treatment course [3,18,19]. Evaluation of soft tissue deformation is challenging also with current onboard CBCT technology and lacks consistent methodological guidelines. Combination of MRI device and linear accelerator may prove useful. The given margins can be updated for the contribution of soft tissue deformations and movements with respect to the bony landmarks when available.

We selected the most important regions that receive the largest doses in the HNC RT. Larger setup errors may occur caudal from C7 corresponding to the level of elective lymph nodes [6]. We excluded larynx since it was not visible in the investigated images. The movement of larynx may be predominantly physiological depending not much on the patient fixation, and the literature data for suitable margins can be applied [6]. Our residual position errors of C1-2 and C5-7 were quite similar to the previously reported values [6]. These structures correspond to the important neck nodal regions 1 and 4. The systematic errors obtained for the occiput bone are close to those reported for bony landmarks near the parotid glands obtained with similar IGRT protocol [20]. Position errors obtained for the mandible, however, were larger than previously reported [6]. This may be partly due to larger movement of the mandible, and partly due to more extreme areas used for the determination of the position errors. In addition, the localization is based on planar projections of the areas suggesting rather maximal than average position errors.

Our experimental 3D margins were consistent with the average of the calculated margins suggesting the validity of van Herk’s formula for our data. The experimentally and theoretically estimated thresholds were consistent within ± 0.5 mm except for the occiput bone and the mandible with the MID_PTV match. Previously thresholds of 4.8 mm [6] and 4 mm [12] have been estimated based on replanning rate of 25% and 33% of the patients, respectively. Slight differences between our and literature results may be explained by different patient immobilization systems, different imaging methods (CBCT vs. 2D kV) and different criterion used to obtain the threshold. In contrast to the reported values, we determined the threshold by retaining 5 mm margins sufficient. Determination of an optimal number of measurements to confirm the systematic nature of the error and the need for replanning was out of the scope of this study, but values from 5 to 8 have been proposed depending on the optimization method [12].

Our results suggest that there are both rotation and shape changes in the vertebra. Rigid rotation of patient was not commonly observed and couch rotation corrections would have only partial benefit. Large random error (σ = 2.5 mm) between the mandible and C1-2 together with a weak correlation between their movements suggest that the mandible may move quite independently in cranio-caudal direction. This movement might be reduced by confirming that the patient bites slightly or by using a mouthpiece [8]. “Nod-of-head” movement was clearly seen in approximately half of the images. A weak correlation between the mandible and the occiput bone movements can be explained by relatively small effect of this movement for the occiput bone. Quite large margins required for the occiput bone in vertical direction may be partly due to uncertainty in the determination of skull base from the orthogonal images. Fortunately, PTVs do not usually extend much towards the occiput bone (see Figure 1b). Our results suggest that time trends of position errors of the bony landmarks are rare consistently with the literature [6]. Such time trends may occur for soft tissues [3,21] further emphasizing the role of regular onboard CBCT verification combined to frequent routine 2D x-ray method.

We excluded investigation of planning organs at risk volumes (PRV). With IMRT technique, 1D, 2D or 3D margins are required depending on the organ at risk and the shape of dose distribution. The PRV margins, however, can be calculated from the results given in Table 1 by using a factor of 1.3, 2.2 or 2.5 for systematic 1D, 2D or 3D errors, respectively [22]. The results obtained for the target can be directly applied for 3D cases.

## Conclusions

We determined setup errors and sufficient setup margins for HNC RT based on routine 2D kV image guidance. The results were obtained for bony landmarks and their appropriate application requires regular 3D verification of the validity of the landmarks for the relevant soft tissues and tumours. The bony landmarks were divided into the four most important subregions. The combined effect of rotation, mutual movement and shape changes of the bony landmarks required approximately two times larger margins than were needed by assuming a rigid target. The margins were dependent on the subregions related to the target volume, frequency of image guidance applied and early correction of clinically important systematic displacements of the relevant subregions. To retain small commonly used margins of 5 mm sufficient for most of the subregions and the 2D online images are matched to the bony structures in the middle of the target volume, daily image guidance is needed and an action level of 4 mm should be applied for the systematic 3D displacement of a subregion. Consistent threshold values were derived with two different methods based on clinical data. When the threshold is exceeded, it is optimal to correct patient fixation first. If this is not successful, confirmation of clinical importance of the displacement may be useful by using 3D onboard imaging in the consideration of potential adaptive replanning. The action level derived was introduced in our clinical practise. The results obtained for the random position errors can be used to evaluate whether fixation should be corrected for an individual patient.

## Competing interests

The authors declare that they have no competing interests.

## Author contributions

MK and ML drafted the manuscript and performed the data analysis. ML performed the data collection. MK, ML and TT participated in the study design and coordination. TW, SP, SH and P-L K-L discussed the outcome during the study. All authors read and approved the final manuscript.
